# Factors associated with long-term opioid use among patients with axial spondyloarthritis or psoriatic arthritis who initiate opioids

**DOI:** 10.1093/rheumatology/keae444

**Published:** 2024-08-16

**Authors:** Yun-Ting Huang, David A Jenkins, Belay Birlie Yimer, Meghna Jani

**Affiliations:** Centre for Epidemiology Versus Arthritis, Centre for Musculoskeletal Research, The University of Manchester, Manchester, UK; Centre for Health Informatics, Division of Informatics, Imaging and Data Science, The University of Manchester, Manchester, UK; Centre for Health Informatics, Division of Informatics, Imaging and Data Science, The University of Manchester, Manchester, UK; Centre for Epidemiology Versus Arthritis, Centre for Musculoskeletal Research, The University of Manchester, Manchester, UK; Centre for Epidemiology Versus Arthritis, Centre for Musculoskeletal Research, The University of Manchester, Manchester, UK; NIHR Manchester Biomedical Research Centre, Manchester University NHS Foundation Trust, Manchester Academic Health Science Centre, Manchester, UK; Department of Rheumatology, Salford Royal Hospital, Northern Care Alliance, Salford, UK

**Keywords:** axial spondyloarthritis, psoriatic arthritis, long-term opioid use, opioids, opiates, risk factors

## Abstract

**Objective:**

Up to one in five patients with axial spondyloarthritis (AxSpA) or psoriatic arthritis (PsA) newly initiated on opioids transition to long-term use within the first year. This study aimed to investigate individual factors associated with long-term opioid use among opioid new users with AxSpA/PsA.

**Methods:**

Adult patients with AxSpA/PsA and without prior cancer who initiated opioids between 2006 and 2021 were included from Clinical Practice Research Datalink Gold, a national UK primary care database. Long-term opioid use was defined as having ≥3 opioid prescriptions issued within 90 days, or ≥90 days of opioid supply, in the first year of follow-up. Individual factors assessed included sociodemographic, lifestyle factors, medication use and comorbidities. A mixed-effects logistic regression model with patient-level random intercept was used to examine the association of individual characteristics with the odds of long-term opioid use.

**Results:**

In total, 10 300 opioid initiations were identified from 8212 patients (3037 AxSpA; 5175 PsA). The following factors were associated with long-term opioid use: being a current smoker (OR: 1.62; 95%CI: 1.38,1.90), substance use disorder (OR: 2.34, 95%CI: 1.05,5.21), history of suicide/self-harm (OR: 1.84; 95%CI: 1.13,2.99), co-existing fibromyalgia (OR: 1.62; 95%CI: 1.11,2.37), higher Charlson Comorbidity Index (OR: 3.61; 95%CI: 1.69,7.71 for high scores), high MME/day at initiation (OR: 1.03; 95%CI: 1.02,1.03) and gabapentinoid (OR: 2.35; 95%CI: 1.75,3.16) and antidepressant use (OR: 1.69; 95%CI: 1.45,1.98).

**Conclusions:**

In AxSpA/PsA patients requiring pain relief, awareness of lifestyle, sociodemographic and prescribing characteristics associated with higher risk of long-term opioid use can prompt timely interventions such as structured medication reviews and smoking cessation to promote safer prescribing and better patient outcomes.

Rheumatology key messages

- Lifestyle measures such as current smoking were associated with long-term opioid use within 1 year after opioid initiation.
- Prescribing factors such as high MME/day at initiation, concurrent gabapentinoids and antidepressants were associated with long-term opioid use.
- Higher social deprivation, substance use disorder, suicide/self-harm, co-existing fibromyalgia, and higher baseline comorbidities were also associated.

## Introduction

Opioids have been an effective class of drugs for acute pain but remain a controversial option for chronic pain management within rheumatic and musculoskeletal diseases (RMDs). Opioid prescribing has increased considerably amongst people living with non-cancer chronic pain between 2006 and 2017 in UK primary care [1]. RMD patients frequently take opioids for pain relief, especially given limited options for pain management. The trends of existing opioid users have increased since 2006 and dropped in 2019 for axial spondyloarthritis (AxSpA) and dropped in 2020 for psoriatic arthritis (PsA) patients [2].

A high proportion of RMD patients transition to long-term opioid use within one year following opioid initiation in the UK [3]. Long-term opioid use has been studied extensively but defined using varying definitions in the literature [4], and is associated with opioid dependence, abuse and harm [5]. Amongst patients who are newly initiated on opioids, up to 1 in 4–5 patients became long-term opioid users, with 23.8% for AxSpA, and 21.6% for PsA [3]. The proportion was much higher than that in the UK general population with non-cancer pain, in which 1 in 7 patients (14.6%) transitioned to a long-term user [1]. In North America, AxSpA was the condition associated with the highest risk of long-term opioid prescriptions amongst patients with RMDs, with a cumulatively high supply of opioids (≥270 days per year) [6, 7]. A German study reported that 9.1% of AxSpA patients transitioned to long-term opioid use and 9.5% for PsA in 2019 [8]. This higher frequency in AxSpA and PsA highlights the complexity of pain management in this patient cohort and the importance of optimising opioid therapy to reduce the risk of long-term use and associated harms.

Most research investigating risk factors associated with long-term opioid use is restricted to post-surgical populations using US administrative data. The evidence on the patients with AxSpA/PsA is scarce. A previous UK study examined analgesic prescribing in patients with inflammatory arthritis in England and reported that opioids are often initiated in the peri-inflammatory arthritis diagnosis period and often continue since started [9]. The effect of disease-modifying anti-rheumatic drugs (DMARDs), opioid dosage and lifestyle factors such as smoking has not been explored. Some medications (e.g. antidepressants) that are often co-prescribed for chronic pain may also influence the duration of opioid therapy and need to be considered.

AxSpA and PsA are some of the most prevalent inflammatory RMDs, in addition to rheumatoid arthritis (RA). They have overlapping disease mechanisms and similar therapeutic options, including non-steroidal anti-inflammatory drugs (NSAIDs), conventional synthetic DMARDs (csDMARDs) and biological therapies. Given the high frequency of long-term opioid use in AxSpA and PsA, there is a pressing need to identify potential factors associated with long-term opioid use in this population using a rigorous approach, which can contribute to personalized treatment and help tailor targeted interventions. Therefore, this study aimed to evaluate potential factors associated with long-term opioid use among AxSpA/PsA patients who were newly initiated on opioids.

## Methods

### Study population

This study included patients aged ≥18 years with a diagnosis of axial spondyloarthritis (AxSpA) or psoriatic arthritis (PsA) and a new episode of opioid use between 1 January 2006 and 31 August 2021 from the Clinical Practice Research Datalink (CPRD) GOLD. CPRD is a database of anonymized UK primary care electronic health records representative of the national population [10]. Opioids prescribed up to 6 months before or any time after an RMD diagnosis were included in this study. A new episode of opioid use was defined as the first opioid prescription after at least two years of opioid-free for an individual (Supplementary Fig. S1, available at *Rheumatology* online). Clinical diagnoses (e.g. AxSpA) were identified using Read Codes and medications were captured using Product Codes. The code lists were generated and checked by researchers with clinical backgrounds. Patients were assigned to either the AxSpA or PsA cohort using the primary diagnosis (i.e. the earliest diagnosis) if having more than one RMD diagnosis, so each cohort was mutually exclusive to another. Individuals with a cancer diagnosis (except non-melanoma skin cancer) five years prior to the new opioid episode were excluded due to a different drug utilization pattern among cancer patients. Those who had less than one year follow-up were also excluded.

### Long-term opioid use

Long-term opioid use was defined as having ≥3 opioid prescriptions issued within a 90-day period, or a sum of ≥90 days of opioid supply, in the first year of follow-up but excluding the first 30 days. The first 30 days are likely to contribute to short-term opioid use often used in acute pain, helping not to overestimate long-term opioid use. This definition has been used most commonly in the literature and will make the study results comparable to previous studies [1, 3].

### Socio-demographic factors

Most socio-demographic characteristics were unchanged over time and unique to each individual, including sex, ethnicity and the Index of Multiple Deprivation (IMD). Sex was collected on the index date of the first new opioid episode. Ethnicity, classified as white *vs* mixed/non-white, was available via the linked data of Hospital Episode Statistics (HES) and Read Codes recorded in CPRD. IMD obtained through data linkage was presented in quintiles, with first quintile as the least deprived and fifth as the most deprived. Age was time-varying and collected on the index date of the new opioid episode. Ethnicity and IMD had a separate category of missingness due to a considerable proportion of missing values.

### Opioids and concurrent medications

Medication use changed over time and was identified for each unique entry (i.e. each new episode of opioid use) for the same individual. Morphine milligram equivalents (MME) per day were collected on the index date of the new episode of opioid prescription. The duration of prescriptions was estimated using a previously published drug preparation algorithm [11, 12], and the sequential decisions are described in Supplementary Table S1, available at *Rheumatology* online. MME/day was determined by converting each opioid’s daily dosage using the equivalent analgesic ratio released by the CDC [13] and then summing the MME/day for all different opioids prescribed on the index date. To define recent exposure to other medications of interest, Product Codes in a one-year look-back period before the new episode of opioid use were identified and presented as a binary variable (i.e. yes *vs* no). The medications included NSAIDs, csDMARDs (i.e. hydroxychloroquine, sulfasalazine, leflunomide, methotrexate), benzodiazepines, gabapentinoids, antipsychotics and antidepressants. Information on biological agents and targeted synthetic small molecules is not available in CPRD as they are prescribed in hospitals in the UK.

### Comorbidity

Comorbidities, including suicide or self-harm (i.e. those who intended to or had committed suicide or self-harm), depression, alcohol dependence, substance use disorder and the Charlson Comorbidity Index (CCI) were assessed using Read Codes in a five-year look-back period before the new opioid episode. The CCI was presented in three categories: low 0, medium (1–3) and high (4+) scores while other diseases were binary (i.e. yes *vs* no). Coexisting fibromyalgia was identified using Read Codes and diagnosed later than the primary diagnosis (i.e. AxSpA or PsA). For each new episode of opioid use, opioids needed to be prescribed after the date six months prior to the first diagnosis of fibromyalgia; otherwise, coexisting fibromyalgia would be coded as zero.

### Lifestyle factors

Lifestyle factors including body mass index (BMI) and smoking status were identified using previously validated algorithms [14–16]. BMI and smoking status were captured within five years of each new opioid episode and the latest record was used for subsequent analysis. BMI was classified into five categories: underweight (<18.5), normal (18.5–24.9), overweight (25–29.9), obese (30–39.9) and morbidly obese (≥40) [17]. Smoking status was categorised as three levels: never smoker, former smoker and current smoker.

### Statistical analysis

A mixed-effects logistic regression model with patient-level random intercept was used to examine the association of different individual characteristics with the odds of transitioning to long-term opioid use. Fitting a patient-level random-effects model captures the dependence of the repeated data within the same patient because each patient may potentially contribute multiple new opioid episodes in the 15-year study period. The mixed-effect model included and adjusted for the listed socio-demographics, medication use, comorbidities and lifestyle factors we introduced above. Adjusted odds ratios (ORs) and 95% confidence intervals (CIs) from the model were reported. As missingness was coded as a separate category, patients with missing values were still included in the analysis. We analysed the cohorts of AxSpA and PsA separately as sensitivity analyses to examine the robustness of the primary analysis. The second sensitivity analysis additionally included an indicator of previous opioid initiation, whilst the third sensitivity analysis included a variable indicating if a patient became a long-term opioid user in previous initiations. Lastly, we conducted a sensitivity analysis by aggregating csDMARDs, including hydroxychloroquine, sulfasalazine, leflunomide and methotrexate, into one group. The statistical analyses were conducted using Stata version 13.1. The ISAC approval number from CPRD is 20_000143.

### Ethics approval

The study was approved by the CPRD’s Independent Scientific Advisory Committee (approval number: 20_000143).

## Results

**Figure 1.**
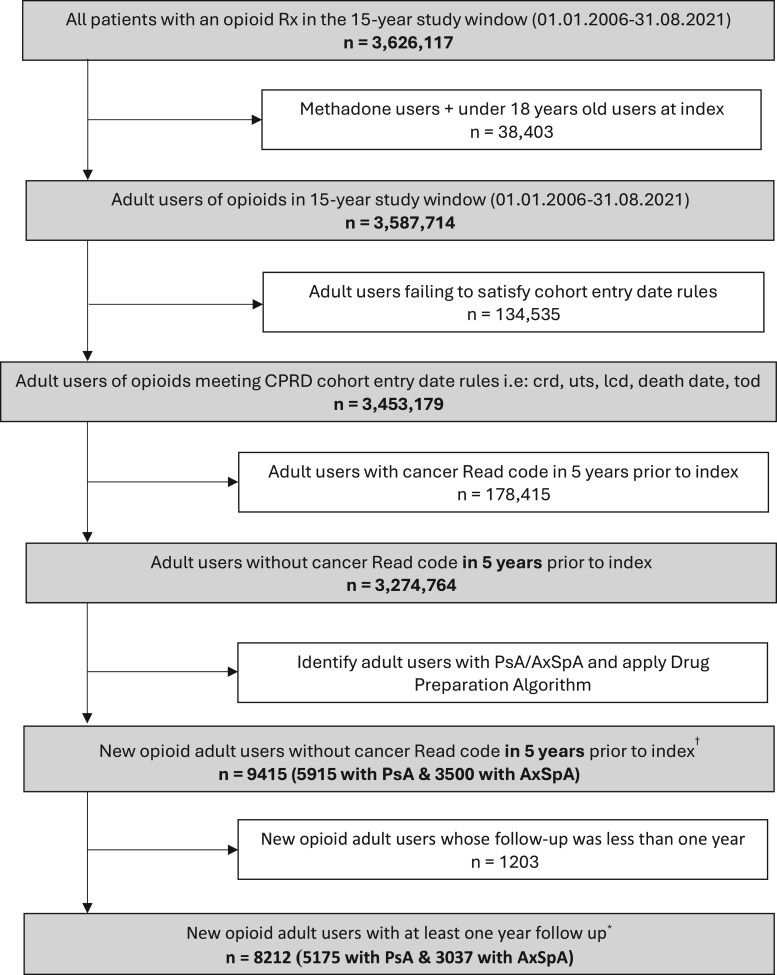
Flowchart for the study cohort derivation. ^a^The number of patients referred to unique patient IDs. A patient could have a new episode of opioid use again when the time window between opioid prescriptions was more than two years. ^b^The follow-up of eligible individuals started with the current registration date that the practice also met data quality metrics (i.e. up to standard date) and ended up with the earliest time of transfer out dates, death dates and last collection dates. For those who took opioids, the start of follow-up also accounted for the time when an individual had an opioid prescription up to 6 months before or anytime after an RMD diagnosis. crd: date the patient’s current period of registration with the practice began; lcd: date of the last collection of data for the practice; hes: hospital episode statistics; tod: date the patient transferred out of the practice; uts: up to standard/date at which the practice data was deemed to be of research quality

This study included 10 300 new episodes of opioid use between 2006 and 2021, from 8212 unique patients. Of the 10 300 opioid initiations, 2310 (22.4%) transitioned to long-term use within one year. The 8212 patients involved 3037 with AxSpA as the primary diagnosis and 5175 with PsA. The derivation of the study cohort is described in Fig. 1 and baseline characteristics are summarized in Table 1. The mean age was similar in AxSpA [mean = 49.0, standard deviation (SD)=14.8] and PsA (mean = 50.4, SD = 14.1) patients, while sex distribution was quite different between the two cohorts, with 29.5% of females in AxSpA and 52.1% in PsA. There was missingness for ethnicity (∼30%) and IMD (∼60%) after the data linkage. The former is not well recorded in primacy care but can be obtained through data linkage to HES; the latter is only available via data linkage. The distribution of ethnicity and social deprivation was similar between AxSpA and PsA. Among the whole cohort including those missing ethnicity, 65.9% were white and 3.0% were mixed or non-white ethnicity. The proportion of patients decreased when social deprivation increased, with 8.6% in the least deprived category and 5.5% in the most deprived category for the entire cohort.

**Table 1.** Baseline characteristics of AxSpA/PsA patients who initiated opioids (*n* = 8212^a^)

	AxSpA (*n* = 3037)	PsA (*n* = 5175)	Entire cohort (*n* = 8212)
**Sociodemographic factors**			
Age, mean (SD)	49.0 (14.8)	50.4 (14.1)	49.9 (14.3)
Sex – Female, *n* (%)	897 (29.5)	2698 (52.1)	3595 (43.8)
Ethnicity, *n* (%)			
White	2045 (67.3)	3367 (65.1)	5412 (65.9)
Mixed/non-white	108 (3.6)	136 (2.6)	244 (3.0)
Missing	884 (29.1)	1672 (32.3)	2556 (31.1)
IMD, *n* (%)			
1 (Least deprived)	295 (9.7)	410 (7.9)	705 (8.6)
2	288 (9.5)	388 (7.5)	676 (8.2)
3	291 (9.6)	376 (7.3)	667 (8.1)
4	239 (7.9)	317 (6.1)	556 (6.8)
5 (Most deprived)	182 (6.0)	271 (5.2)	453 (5.5)
Missing	1742 (57.4)	3413 (66.0)	5155 (62.8)
**Medication use**			
Index MME/day, median (IQR)	24.9 (13.5–27.0)	21.4 (9.6–27.0)	22.5 (10.0–27.0)
NSAIDs use, *n* (%)	1973 (65.0)	3205 (61.9)	5178 (63.1)
Hydroxychloroquine use, *n* (%)	12 (0.4)	36 (0.7)	48 (0.6)
Sulfasalazine use, *n* (%)	155 (5.1)	605 (11.7)	760 (9.3)
Leflunomide use, *n* (%)	4 (0.1)	166 (3.2)	170 (2.1)
Methotrexate use, *n* (%)	90 (3.0)	1258 (24.3)	1348 (16.4)
Benzodiazepine use, *n* (%)	268 (8.8)	369 (7.1)	637 (7.8)
Gabapentinoid use, *n* (%)	93 (3.1)	163 (3.2)	256 (3.1)
Antipsychotic use, *n* (%)	109 (3.6)	186 (3.6)	295 (3.6)
Antidepressant use, *n* (%)	572 (18.8)	1132 (21.9)	1704 (20.8)
**Comorbidities**			
History of suicide/self-harm, *n* (%)	37 (1.2)	84 (1.6)	121 (1.5)
History of depression, *n* (%)	376 (12.4)	710 (13.7)	1086 (13.2)
History of alcohol dependence, *n* (%)	42 (1.4)	88 (1.7)	130 (1.6)
History of substance use disorder, *n* (%)	24 (0.8)	19 (0.4)	43 (0.5)
Co-existing fibromyalgia, *n* (%)	46 (1.5)	107 (2.1)	153 (1.9)
CCI, *n* (%)			
Low 0	2466 (81.2)	4115 (79.5)	6581 (80.1)
Medium (1–3)	552 (18.2)	1034 (20.0)	1586 (19.3)
High (4+)	19 (0.6)	26 (0.5)	45 (0.6)
**Lifestyle factors**			
BMI, *n* (%)			
Underweight	31 (1.0)	31 (0.6)	62 (0.8)
Normal	685 (22.6)	802 (15.5)	1487 (18.1)
Overweight	746 (24.6)	1207 (23.3)	1953 (23.8)
Obese	505 (16.6)	1271 (24.6)	1776 (21.6)
Morbidly obese	48 (1.6)	233 (4.5)	281 (3.4)
Missing	1022 (33.7)	1631 (31.5)	2653 (32.3)
Smoking status, *n* (%)			
Never	1131 (37.2)	2183 (42.2)	3314 (40.4)
Former	853 (28.1)	1553 (30.0)	2406 (29.3)
Current	792 (26.1)	1033 (20.0)	1825 (22.2)
Missing	261 (8.6)	406 (7.9)	667 (8.1)

a This study included 8212 unique patients, resulting in 10 300 new opioid episodes over a 15-year follow-up. A patient might initiate opioid use more than once. Medications, comorbidities and lifestyle factors were evaluated each time a patient started opioid treatment. This table only included the first episode of opioid initiation for each patient to avoid changing demographic features’ distribution, which remained unchanged over time.

Medication use was similar between AxSpA and PsA but some differences were observed. A higher proportion of PsA patients were observed to be prescribed sulfasalazine (11.7%), leflunomide (3.2%) and methotrexate (24.3%) compared with AxSpA patients (5.1%, 0.1% and 3.0%, respectively), in line with current guidelines [18, 19]. Both cohorts had up to one in five prescribed antidepressants, with 18.8% for AxSpA and 21.9% for PsA. AxSpA and PsA cohorts showed a similar prevalence of relevant comorbidities but had different patterns in lifestyle factors. PsA patients had a higher proportion of obesity (24.6%) and morbid obesity (4.5%) than AxSpA patients (16.6% and 1.6%, respectively). AxSpA cohort instead showed a higher proportion of current smokers (26.1%) compared with PsA (20.0%). Supplementary Table S2, available at *Rheumatology* online, additionally presented proportions of patients stratified by transitioning to long-term opioid use or not for some features re-evaluated each time a patient initiated opioid treatment.

**Figure 2.**
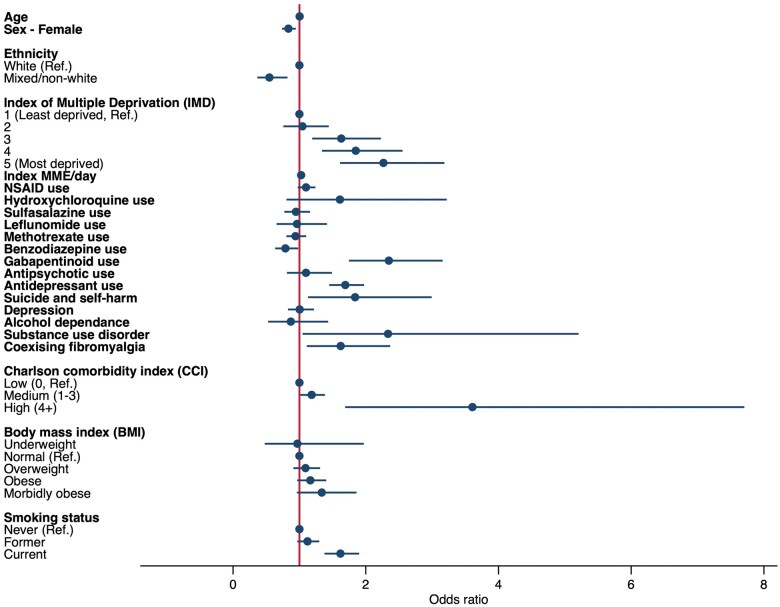
Factors associated with long-term opioid use within one year after initiation for AxSpA and PsA. Factors including socio-demographics, medication use, comorbidities and lifestyle factors were included in a mixed-effects logistic regression model. Point estimates were adjusted odds ratios and horizontal lines referred to 95% CIs

**Figure 3.**
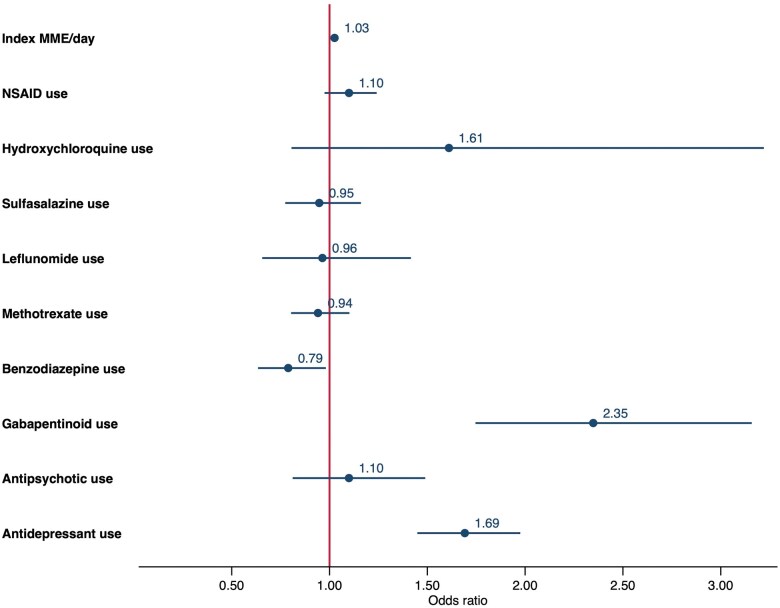
Medications associated with long-term opioid use within one year after initiation for AxSpA and PsA (a subset of Fig. 2). Factors including socio-demographics, medication use, comorbidities and lifestyle factors were included in a mixed-effects logistic regression model. Point estimates were adjusted odds ratios and horizontal lines referred to 95% CIs

After accounting for individual-level variations and comprehensive factors ranging from socio-demographics, medication use and comorbidities to lifestyle factors, socioeconomic deprivation was associated with an incremental higher risk of long-term opioid use. In comparison with the least deprived quintile, the most deprived area (fifth quintile) was associated with a doubling of risk (OR = 2.27, 95% CI = 1.61, 3.19); fourth quintile with 85% increase (OR = 1.85, 95% CI = 1.34, 2.55); third quintile with 63% increase (OR = 1.63, 95% CI = 1.19, 2.23) (Fig. 2). The details of ORs and 95% CI are presented separately in Supplementary Table S3, available at *Rheumatology* online. AxSpA and PsA patients who had a history of substance use disorder (OR = 2.34, 95% CI = 1.05, 5.21) and a history of suicide or self-harm (OR = 1.84, 95% CI = 1.13, 2.99) and coexisting fibromyalgia (OR = 1.62, 95% CI = 1.11, 2.37) were more likely to become long-term opioid users within one year after an opioid initiation. Patients with higher CCI scores were associated with higher odds of the outcome: medium score (1–3) (OR = 1.18, 95% CI = 1.01, 1.39) and high scores (≥4) (OR = 3.61, 95%CI = 1.69, 7.71). Current smokers were observed to have a 62% increase in the risk of becoming long-term users (OR = 1.62, 95%CI = 1.38, 1.90) compared with those who never smoked. As shown in Fig. 3, each unit increase in MME/day, which represented a starting dose of all opioid treatments, led to a 3% increase in the risk of long-term use (OR = 1.03, 95% CI = 1.02, 1.03). Medication use including gabapentinoids (OR = 2.35, 95% CI = 1.75, 3.16) and antidepressants (OR = 1.69, 95% CI = 1.45, 1.98) was also associated with an increased risk.

Some individual factors were associated with reduced risk of long-term opioid use, including being female (OR = 0.83, 95% CI = 0.74, 0.95), taking benzodiazepines (OR = 0.79, 95% CI = 0.63, 0.98) and being mixed or non-white ethnicity (OR = 0.55, 95% CI = 0.37, 0.82) (Fig. 1). The association with benzodiazepines only became significant after accounting for antidepressant use. Medications that may help modulate inflammatory and immune responses for AxSpA and PsA, such as NSAIDs and csDMARDs (e.g. methotrexate), were not significantly associated with long-term opioid use. Being a former smoker and BMI were not associated with higher risk of the outcome.

The findings of sensitivity analysis were similar to the primary results (Supplementary Table S4, available at *Rheumatology* online). The lower risk of long-term opioid use associated with being female (OR = 0.84, 95% CI = 0.71, 1.00) and benzodiazepine use (OR = 0.67, 95% CI = 0.48, 0.92) was only observed in PsA patients. When including an additional indicator of previous opioid initiation, most of the results were similar to the primary results (Supplementary Fig. S2, available at *Rheumatology* online). Having prior experience in initiating opioids was associated with a lower risk of long-term use (OR = 0.56, 95% CI = 0.48, 0.65). This risk further reduced (OR = 0.15, 95% CI = 0.07, 0.31) if a patient had transitioned to a long-term opioid user in previous opioid initiations (Supplementary Fig. S3, available at *Rheumatology* online). The use of csDMARDs remained not significantly related to long-term opioid use, despite different csDMARDs aggregated into one category (Supplementary Fig. S4, available at *Rheumatology* online).

## Discussion

This study identified individual factors associated with long-term opioid use amongst patients with AxSpA or PsA, ranging from sociodemographic to pharmacological treatments, comorbidities and lifestyle factors. After accounting for individual-level variations and the listed potential factors, socioeconomic deprivation, a high MME/day at opioid initiation, the use of gabapentinoids and antidepressants, a history of substance use disorder and suicide/self-harm, coexisting fibromyalgia, multimorbidity and being a current smoker were associated with long-term opioid use. We did not observe significant associations with the underlying treatments of AxSpA and PsA, specifically csDMARDs, antipsychotic use, a history of depression and BMI.

### Comparison with existing literature

Smoking has been shown to be associated with poor treatment response and worse outcomes (e.g. cardiovascular comorbidity) in both PsA and AxSpA [20–22]. We found current smokers were more likely to transition to long-term use. A possible mechanism is that smoking lowers response to medications such as tumour necrosis factor inhibitors (TNFi) treatments [21, 22], which may lead to increased disease activity and higher pain scores, requiring more long-term opioid use. Smokers have also been found to consume greater quantities of opioids in people with chronic pain [23] and postoperative patients [24]. There is some evidence that co-treatment of smoking and opioid use may lead to better outcomes in those seeking treatment for opioid addiction [25]. A former smoking history showed no association in our study, slightly different from previous findings of past or current nicotine use being at high risk of long-term use among new opioid users [23]. Also, complex comorbidities may interact with the underlying PsA/AxSpA and interfere with the disease control [26–28], leading to an increased probability of prolonged opioid use.

Higher social deprivation, assessed by either Townsend score, IMD or other socio-economic indicators (e.g. low income) has been associated with a higher risk of long-term use in chronic pain populations with or without cancer [1, 29] and people living with inflammatory arthritis [9]. A higher deprivation was related to lower health literacy, and the difficulty of understanding information might make patients less willing to discontinue opioids or communicate with their general practitioners (GPs) ineffectively, resulting in prolonged use of opioid therapy [30]. A higher opioid dosage (i.e. MME/day) at initiation, gabapentinoid use, suicide/self-harm, substance use disorder, and fibromyalgia demonstrate a strong and consistent relationship with long-term opioid use in different populations [1, 9, 23, 31–34] and our cohort. Sensitivity analyses found that prior experience with initiating opioids and transitioning to long-term use was associated with a lower risk of long-term use. The findings likely reflect the increased caution and efforts made to prevent patients with previous long-term opioid use from returning to the same situation. This result may not be directly comparable to previous research on preoperative opioid use, which is related to higher odds of long-term postoperative opioid treatments [31–33], due to our definition of new opioid episodes requiring at least two years of being opioid-free.

Among PsA and AxSpA patients, antidepressant use was associated with an increased risk. A bidirectional relationship between depression and pain has been shown in the literature, but a history of depression was no longer related to long-term opioid use. Patients with depression often suffer from chronic pain so may take antidepressants and opioids concurrently [35]. Antidepressants are often co-prescribed with opioid therapy because they are recommended for chronic primary pain management by the latest National Institute for Health and Care Excellence (NICE) guideline [36]. Antidepressants in combination with opioids have some concerns about an increased risk of serotonin toxicity and less pain relief achieved [37, 38]. The combination therapy may also represent a high level of pain that requires longer analgesic treatments, subsequently leading to long-term opioid use.

Being a woman has been reported to be a consistent risk factor for long-term opioid use in both general and surgical populations [1, 31–33], whereas women were less likely to become long-term opioid users amongst AxSpA and PsA patients. The prevalence of PsA is considered equal between men and women, while AxSpA is more frequently diagnosed in men compared with women (3:1), as shown in our study [39, 40]. The lower risk observed in women, however, is undermined if separating the two conditions, likely because of the reduced sample size. No direct comparison with the previous study on inflammatory arthritis can be made because they did not include sex in the multivariable model [9]. Mixed or non-white ethnicity consistently shows a lower risk of long-term opioid use in the non-cancer chronic pain population [1], nursing home residents [41] and our study.

Obesity is also believed to affect treatment response in PsA and AxSpA by inducing a chronic low-grade inflammatory state through inflammatory mediators such as TNF-alpha and interleukin-6 [42, 43]. It can also change the pharmacokinetics of anti-TNF agents and other biologics and reduce clinical response [43], leading to increased opioid intake amongst obese people. A previous study on fibromyalgia found severely obese patients were more likely to become long-term users [34]. BMI, however, showed no association with long-term opioid use for PsA and AxSpA in our study.

### Strength and limitations

This study, to our knowledge, is the first to examine factors associated with long-term opioid use among patients with PsA and AxSpA, which adjusts for csDMARDs, lifestyle factors and concurrent pain relievers. We included a comprehensive list of medications and assessed medication use within one year before each opioid initiation. We also evaluated comorbidities for each opioid initiation but included a longer look-back period (i.e. five years) as comorbidities tend to be chronic with codes not repeatedly entered once diagnosed, as opposed to changes in medications, which are more dynamic. We used a time-varying approach to ensure a more accurate assessment of concurrent medications and comorbidities, which are very likely to influence the likelihood of long-term opioid use.

A number of limitations need to be considered in interpreting the findings. Information on opioids prescribed in the hospital or obtained over the counter is not captured in CPRD and in primary care records. Furthermore, some information is lacking in primary care data, including biological treatments, which are prescribed in hospitals in the UK, and disease activity and pain severity scores that are not routinely recorded in primary care.

### Implications for clinical practice

This study highlights the importance of smoking behaviour in patients with AxSpA or PsA, in which current smokers were more likely to transition to long-term opioid use, possibly due to the mechanisms highlighted earlier. Higher social deprivation, substance use disorder, suicide or self-harm, fibromyalgia, and higher CCI scores were also associated with long-term opioid use. An increased risk of high MME/day at initiation and concurrent use of gabapentinoids and antidepressants with long-term opioid use emphasizes the importance of medication profiles in prolonged opioid therapy in this population. Our findings reflect the complexity of chronic pain management among AxSpA/PsA patients, influenced by treatment response to the underlying disease, pain severity due to AxSpA/PsA or other comorbidities (e.g. fibromyalgia) and concurrent use of multiple pain relievers. Awareness of one or more of these patient factors would allow the delivery of more tailored approaches for pain management in clinical practice. These may include early access to non-pharmacological treatments such as regular physiotherapy appointments, access to smoking cessation services, and structured medication reviews in primary care. This study highlights the importance of a comprehensive assessment ranging from health conditions and medication profiles to lifestyle habits for AxSpA/PsA, to help personalize treatment approaches and potentially reduce the risk of unnecessary harms associated with long-term opioid use.

## Conclusion

AxSpA and PsA patients who were current smokers and experienced higher social deprivation, substance use disorder, suicide/self-harm, fibromyalgia and higher CCI scores were more likely to transition to long-term opioid use within one year after opioid initiation. High MME/day at initiation and concurrent use of gabapentinoids and antidepressants were associated with long-term opioid use while underlying treatments of AxSpA and PsA (e.g. csDMARDs) and a history of depression showed no association. Awareness of one or more of these patient factors can prompt tailored approaches for pain management in practice, including non-pharmacological treatments, smoking cessation services and structured medication reviews. This study highlights the importance of a comprehensive assessment, from health conditions and medication profiles to lifestyle habits for AxSpA/PsA, to help personalize treatment approaches and then promote safer prescribing and improve long-term patient outcomes.

## Supplementary material

Supplementary material is available at *Rheumatology* online.

## Supplementary Material

keae444_Supplementary_Data

## Data Availability

This study used pseudonymized patient-level data from the CPRD. To protect patient confidentiality, we cannot publish patient-level data. Other researchers can use patient-level CPRD data in a secure environment by applying to the CPRD Independent Scientific Advisory Committee. Details of the application process and conditions of access are provided by the CPRD at https://www.cprd.com/data-access.
