# Supplementary material for: Factors associated with long-term opioid use among patients with axial spondyloarthritis or psoriatic arthritis who initiate opioids
Source: Rheumatology (Oxford). 2024 Aug 16;64(4):1844–52. doi: 10.1093/rheumatology/keae444 (PMC11962957; doi:10.1093/rheumatology/keae444)
Supplement: keae444_Supplementary_Data [file keae444_supplementary_data.docx]

**Supplementary materials**

**Figure S1 Definition of the new episode (initiation) of opioid prescription**

≥ 2 years

≥ 2 years

1-year follow up to define long-term use

Opioid Rx

New episode

New episode

**Figure S2 Sensitivity analysis for factors associated with long-term opioid use in AxSpA and PsA by including an additional variable of previous opioid initiation.**

**Figure S3 Sensitivity analysis for factors associated with long-term opioid use in AxSpA and PsA by including an additional variable indicating if a patient became a long-term opioid user in previous initiations.**

**Figure S4 Sensitivity analysis for factors associated with long-term opioid use in AxSpA and PsA using an aggregated variable of csDMARDs^*^.**

**^*^** Conventional synthetic DMARDs (csDMARDs) included hydroxychloroquine, sulfasalazine, leflunomide, and methotrexate.

**Table S1 Decisions made in the drug preparation algorithm**

| Decision node | Description |
| --- | --- |
| Decision 1: Handle implausible qty^*^ | 1c1 set to mean for individual's prescriptions for that drug |
| Decision 2: Handle missing qty^*^ | 2b1 set to mean for individual's prescriptions for that drug |
| Decision 3: Handle implausible ndd^*^ | 3c1 set to mean for individual's prescriptions for that drug |
| Decision 4: Handle missing ndd^*^ | 4b3 set to mean for populations's prescriptions for that drug |
| Decision 5: Clean durations that are longer than clinically plausible | 5c_6 set to 6 months if > 6 months |
| Decision 6: Select which stop date to use | 6d_30 use mean if gap < 30 days |
| Decision 7: Handle missing stop dates | 7b use mean for that drug for that individual |
| Decision 8: Handle multiple prescriptions for same product on same day | 8b use mean ndd and mean length |
| Decision 9: Handle overlapping prescriptions | 9a do nothing: allow prescriptions to overlap |
| Decision 10: Handle sequential prescriptions with short gaps | 10a do nothing: allow gap |

^*^ qty=total number of things prescribed; ndd=number of things taken per day

**Table S2 Characteristics evaluated each time a patient initiated opioids (n =** **10,300^*^), by transitioning to long-term opioid use or not.**

|  | **No long-term use**  **(n=7990, 77.6%)** | **Long-term use**  **(n=2310, 22.4%)** |
| --- | --- | --- |
| **Medication use** |  |  |
| Index MME/day, median (IQR) | 20 (9.6-27) | 27 (13.5-27.9) |
| NSAIDs use, n (%) | 4963 (62.1) | 1434 (62.1) |
| Hydroxychloroquine use, n (%) | 45 (0.6) | 18 (0.8) |
| Sulfasalazine use, n (%) | 742 (9.3) | 206 (8.9) |
| Leflunomide use, n (%) | 190 (2.4) | 53 (2.3) |
| Methotrexate use, n (%) | 1399 (17.5) | 368 (15.9) |
| Benzodiazepine use, n (%) | 651 (8.2) | 186 (8.1) |
| Gabapentinoid use, n (%) | 195 (2.4) | 155 (6.7) |
| Antipsychotic use, n (%) | 275 (3.4) | 102 (4.4) |
| Antidepressant use, n (%) | 1555 (19.5) | 689 (29.8) |
| **Comorbidities** |  |  |
| History of suicide and self-harm, n (%) | 71 (0.9) | 52 (2.3) |
| History of depression, n (%) | 823 (10.3) | 315 (13.6) |
| History of alcohol dependence, n (%) | 98 (1.2) | 37 (1.6) |
| History of substance use disorder, n (%) | 21 (0.3) | 24 (1.0) |
| Co-existing fibromyalgia, n (%) | 134 (1.7) | 83 (3.6) |
| CCI, n (%) |  |  |
| Low (0) | 6667 (83.4) | 1864 (80.7) |
| Medium (1-3) | 1298 (16.3) | 425 (18.4) |
| High (4+) | 25 (0.3) | 21 (0.9) |
| **Lifestyle factors** |  |  |
| BMI, n (%) |  |  |
| Underweight | 56 (0.7) | 16 (0.7) |
| Normal | 1442 (18.1) | 391 (16.9) |
| Overweight | 1923 (24.1) | 543 (23.5) |
| Obese | 1765 (22.1) | 553 (23.9) |
| Morbidly obese | 261 (3.3) | 98 (4.2) |
| Missing | 2543 (31.8) | 709 (30.7) |
| Smoking status, n (%) |  |  |
| Never | 3457 (43.3) | 767 (33.2) |
| Former | 2513 (31.5) | 656 (28.4) |
| Current | 1619 (20.3) | 596 (25.8) |
| Missing | 401 (5.0) | 291 (12.6) |

^*^ 10,300 new opioid episodes were contributed by 8,212 unique patients.

**Table S3 Model estimates for factors associated with long-term opioid use in AxSpA and PsA^*^.**

| **Factor** | **OR (95% CI)** | **P** |
| --- | --- | --- |
| Age | 1.00 (1.00, 1.01) | 0.128 |
| Sex – Female (Ref. = Male) | 0.83 (0.74, 0.95) | **0.004** |
| Ethnicity – Mixed/non-white (Ref. = White) | 0.55 (0.37, 0.82) | **0.004** |
| IMD (Ref. = 1, Least deprived) |  |  |
| 2 | 1.05 (0.76, 1.44) | 0.786 |
| 3 | 1.63 (1.19, 2.23) | **0.002** |
| 4 | 1.85 (1.34, 2.55) | **<0.001** |
| 5 (Most deprived) | 2.27 (1.61, 3.19) | **<0.001** |
| Index MME/day | 1.03 (1.02, 1.03) | **<0.001** |
| NSAIDs use | 1.10 (0.97, 1.24) | 0.124 |
| Hydroxychloroquine use | 1.61 (0.81, 3.22) | 0.178 |
| Sulfasalazine use | 0.95 (0.77, 1.16) | 0.602 |
| Leflunomide use | 0.96 (0.66, 1.42) | 0.850 |
| Methotrexate use | 0.94 (0.80, 1.10) | 0.449 |
| Benzodiazepine use | 0.79 (0.63, 0.98) | **0.033** |
| Gabapentinoid use | 2.35 (1.75, 3.16) | **<0.001** |
| Antipsychotic use | 1.10 (0.81, 1.49) | 0.541 |
| Antidepressant use | 1.69 (1.45, 1.98) | **<0.001** |
| History of suicide/self-harm | 1.84 (1.13, 2.99) | **0.014** |
| History of depression | 1.00 (0.83, 1.22) | 0.968 |
| History of alcohol dependence | 0.87 (0.53, 1.43) | 0.586 |
| History of substance use disorder | 2.34 (1.05, 5.21) | **0.038** |
| Co-existing fibromyalgia | 1.62 (1.11, 2.37) | **0.012** |
| CCI (Ref. = Low (0)) |  |  |
| Medium (1-3) | 1.18 (1.01, 1.39) | **0.035** |
| High (4+) | 3.61 (1.69, 7.71) | **0.001** |
| BMI (Ref. = Normal) |  |  |
| Underweight | 0.97 (0.48, 1.97) | 0.931 |
| Overweight | 1.09 (0.91, 1.31) | 0.349 |
| Obese | 1.16 (0.97, 1.40) | 0.110 |
| Morbidly obese | 1.34 (0.96, 1.86) | 0.084 |
| Smoking status (Ref. = Never) |  |  |
| Former | 1.12 (0.97, 1.30) | 0.124 |
| Current | 1.62 (1.38, 1.90) | **<0.001** |

A p-value of less than 0.05 was considered significant and highlighted in bold.

**^*^** The variance of the patient cluster is 1.20 (95% CI=0.72, 2.01)

**Table S4 Sensitivity analysis for factors associated with long-term opioid use in AxSpA and PsA separately.**

|  | **AxSpA (n=3780**^*^**)** | | **PsA (n=6520**^*^**)** | |
| --- | --- | --- | --- | --- |
| **Factor** | **OR (95% CI)** | **P** | **OR (95% CI)** | **P** |
| Age | 1.00 (1.00, 1.01) | 0.457 | 1.00 (1.00, 1.01) | 0.140 |
| Sex – Female (Ref. = Male) | 0.87 (0.71, 1.05) | 0.147 | 0.84 (0.71, 1.00) | 0.051 |
| Ethnicity – Mixed/non-white (Ref. = White) | 0.46 (0.26, 0.83) | **0.010** | 0.67 (0.38, 1.19) | 0.169 |
| IMD (Ref. = 1, Least deprived) |  |  |  |  |
| 2 | 1.16 (0.76, 1.78) | 0.494 | 0.95 (0.59, 1.54) | 0.837 |
| 3 | 1.54 (1.01, 2.33) | **0.043** | 1.67 (1.04, 2.67) | **0.032** |
| 4 | 1.78 (1.16, 2.73) | **0.008** | 1.93 (1.19, 3.13) | **0.008** |
| 5 (Most deprived) | 2.09 (1.32, 3.32) | **0.002** | 2.51 (1.52, 4.15) | **<0.001** |
| Index MME/day | 1.02 (1.02, 1.03) | **<0.001** | 1.03 (1.02, 1.03) | **<0.001** |
| NSAIDs use | 1.07 (0.90, 1.28) | 0.426 | 1.11 (0.94, 1.31) | 0.226 |
| Hydroxychloroquine use | 0.54 (0.14, 2.07) | 0.365 | 2.35 (0.97, 5.73) | 0.060 |
| Sulfasalazine use | 1.16 (0.80, 1.68) | 0.431 | 0.87 (0.67, 1.13) | 0.303 |
| Leflunomide use | 3.00 (0.54, 16.84) | 0.212 | 0.94 (0.61, 1.46) | 0.788 |
| Methotrexate use | 1.35 (0.84, 2.16) | 0.221 | 0.92 (0.76, 1.11) | 0.384 |
| Benzodiazepine use | 0.91 (0.68, 1.22) | 0.530 | 0.67 (0.48, 0.92) | **0.015** |
| Gabapentinoid use | 1.94 (1.27, 2.97) | **0.002** | 2.88 (1.88, 4.42) | **<0.001** |
| Antipsychotic use | 1.22 (0.79, 1.88) | 0.375 | 1.00 (0.65, 1.54) | 0.999 |
| Antidepressant use | 1.64 (1.31, 2.07) | **<0.001** | 1.78 (1.43, 2.21) | **<0.001** |
| History of suicide/self-harm | 1.61 (0.75, 3.44) | 0.222 | 2.06 (1.06, 4.01) | **0.033** |
| History of depression | 1.00 (0.75, 1.34) | 0.977 | 1.00 (0.76, 1.31) | 0.997 |
| History of alcohol dependence | 1.02 (0.49, 2.14) | 0.957 | 0.79 (0.40, 1.60) | 0.517 |
| History of substance use disorder | 1.43 (0.55, 3.74) | 0.465 | 4.37 (1.13, 16.82) | **0.032** |
| Co-existing fibromyalgia | 2.48 (1.33, 4.62) | **0.004** | 1.38 (0.83, 2.28) | 0.213 |
| CCI (Ref. = Low (0)) |  |  |  |  |
| Medium (1-3) | 1.27 (1.01, 1.59) | **0.045** | 1.11 (0.89, 1.38) | 0.375 |
| High (4+) | 2.03 (0.68, 6.11) | 0.205 | 6.25 (2.07, 18.89) | **0.001** |
| BMI (Ref. = Normal) |  |  |  |  |
| Underweight | 1.00 (0.39, 2.58) | 0.992 | 0.92 (0.32, 2.68) | 0.883 |
| Overweight | 1.18 (0.92, 1.52) | 0.191 | 1.02 (0.78, 1.34) | 0.890 |
| Obese | 1.25 (0.95, 1.64) | 0.109 | 1.12 (0.86, 1.47) | 0.392 |
| Morbidly obese | 1.61 (0.88, 2.94) | 0.120 | 1.22 (0.79, 1.87) | 0.377 |
| Smoking status (Ref. = Never) |  |  |  |  |
| Former | 1.24 (1.00, 1.55) | 0.055 | 1.07 (0.87, 1.31) | 0.520 |
| Current | 2.05 (1.63, 2.57) | **<0.001** | 1.34 (1.06, 1.69) | **0.014** |

A p-value of less than 0.05 was considered significant and highlighted in bold.

^*^ This study included 8,212 unique patients, with 3,037 AxSpA and 5,175 PsA patients. A patient might initiate opioid use more than once. These patients resulted in 10,300 new opioid episodes, 3,780 in AxSpA and 6,520 in PsA, over a 15-year follow-up.
